# Pharmacological and non‐pharmacological interventions to enhance sleep in mild cognitive impairment and mild Alzheimer's disease: A systematic review

**DOI:** 10.1111/jsr.13229

**Published:** 2020-12-02

**Authors:** Jonathan Blackman, Marta Swirski, James Clynes, Sam Harding, Yue Leng, Elizabeth Coulthard

**Affiliations:** ^1^ North Bristol NHS Trust Bristol UK; ^2^ Bristol Medical School University of Bristol Bristol UK; ^3^ University of Bath Bath UK; ^4^ Department of Psychiatry University of California San Francisco CA USA

**Keywords:** AD, Alzheimer's dementia, Alzheimer's disease, MCI, mild cognitive impairment, sleep

## Abstract

Suboptimal sleep causes cognitive decline and probably accelerates Alzheimer's Disease (AD) progression. Several sleep interventions have been tested in established AD dementia cases. However early intervention is needed in the course of AD at Mild Cognitive Impairment (MCI) or mild dementia stages to help prevent decline and maintain good quality of life. This systematic review aims to summarize evidence on sleep interventions in MCI and mild AD dementia. Seven databases were systematically searched for interventional studies where ≥ 75% of participants met diagnostic criteria for MCI/mild AD dementia, with a control group and validated sleep outcome measures. Studies with a majority of participants diagnosed with Moderate to Severe AD were excluded. After removal of duplicates, 22,133 references were returned in two separate searches (August 2019 and September 2020). 325 full papers were reviewed with 18 retained. Included papers reported 16 separate studies, total sample (*n* = 1,056), mean age 73.5 years. 13 interventions were represented: Cognitive Behavioural Therapy – Insomnia (CBT‐I), A Multi‐Component Group Based Therapy, A Structured Limbs Exercise Programme, Aromatherapy, Phase Locked Loop Acoustic Stimulation, Transcranial Stimulation, Suvorexant, Melatonin, Donepezil, Galantamine, Rivastigmine, Tetrahydroaminoacridine and Continuous Positive Airway Pressure (CPAP). Psychotherapeutic approaches utilising adapted CBT‐I and a Structured Limbs Exercise Programme each achieved statistically significant improvements in the Pittsburgh Sleep Quality Index with one study reporting co‐existent improved actigraphy variables. Suvorexant significantly increased Total Sleep Time and Sleep Efficiency whilst reducing Wake After Sleep Onset time. Transcranial Stimulation enhanced cortical slow oscillations and spindle power during daytime naps. Melatonin significantly reduced sleep latency in two small studies and sleep to wakefulness transitions in a small sample. CPAP demonstrated efficacy in participants with Obstructive Sleep Apnoea. Evidence to support other interventions was limited. Whilst new evidence is emerging, there remains a paucity of evidence for sleep interventions in MCI and mild AD highlighting a pressing need for high quality experimental studies exploring alternative sleep interventions.

## 1 INTRODUCTION

The association between Alzheimer's Disease [AD] and sleep disturbance is well established (Mander, 2013; Tranah et al., 2011). The traditional view has been that AD causes sleep impairment and the extent of symptomatic sleep disturbance correlates with the severity of dementia (Benca et al., 1992; Montplaisir et al., 1995; Pat‐Horenczyk et al., 1998; Prinz et al., 1982; Weldemichael & Grossberg, 2010). Circadian rhythm disorders also contribute to sleep disturbance and worsen with age and AD, possibly related to alterations to the Suprachiasmatic Nucleus secretion of melatonin (Swaab et al., 1985).

A recent prominent theory postulates a bidirectional relationship between poor sleep and AD (Mander et al., 2016) – as well as AD causing sleep disturbance, sleep disturbance may lead directly to pathological accumulation of proteins that cause neurodegeneration (Fultz et al., 2019; Hahn et al., 2014). Beta amyloid is one of the two pathognomonic changes of AD. Levels of soluble Amyloid Beta fluctuate diurnally in both mice and humans showing the crucial role that sleep plays in its clearance (Huang et al., 2012). Amyloid Beta 42 levels have been shown to be significantly increased after a single night of sleep deprivation in healthy adults (Ooms et al., 2014) and slow oscillations are temporally linked to CSF flow (Fultz et al., 2019). Chronic sleep deprivation accelerates accumulation of soluble beta amyloid into insoluble amyloid plaques in two mouse models (Kang et al., 2009). A cascade is envisaged in which poor sleep disrupts the clearance of soluble amyloid, leading to plaque deposition. This precipitates further plaque formation through recognised positive feedback loops (Mandrekar‐Colucci et al., 2012). Regardless of whether sleep disturbance arises directly from accumulation of AD pathology or through an independent problem such as sleep apnoea (Ancoli‐Israel, 2000; Bliwise, 1999; Van Cauter et al., 2000), poor sleep might be a significant contributory factor in the eventual onset and progression of AD.

AD pathology progresses for 1–2 decades before a full dementia diagnosis giving plenty of time for factors such as sleep disturbance to affect the rate of pathological progression (Jack et al., 2010). Therefore, the optimal opportunity to reverse or halt cognitive decline caused by AD pathology, thus maintaining quality of life, will be early in the course of disease – at the Mild Cognitive Impairment or mild AD stage. Early diagnosis utilising molecular biomarkers to accurately pinpoint the cause of cognitive symptoms is becoming more common, most frequently for identification of early/prodromal AD (Sutphen et al., 2014) and this provides an opportunity for targeted intervention.

Disruption of sleep occurs early in AD (Ju et al., 2013; Lim et al., 2013; Sprecher et al., 2017), even before symptoms arise, however, the focus of sleep intervention reviews to date have largely been at the stage of established AD dementia (Mitolo et al., 2018; O'Caoimh et al., 2019) when pathology may be too advanced to change. Sleep disorders are also common in this population affecting up to 60% of patients in memory clinics (Littlejohn et al., 2014). Furthermore, sleep deprivation brings a range of other metabolic and cardiovascular impairments that mean improving sleep is likely to be broadly beneficial in the memory clinic population over and above the potential to modify progression of AD. However, there are no standard sleep treatment protocols within memory clinics and other dementia services.

**Figure 1:**
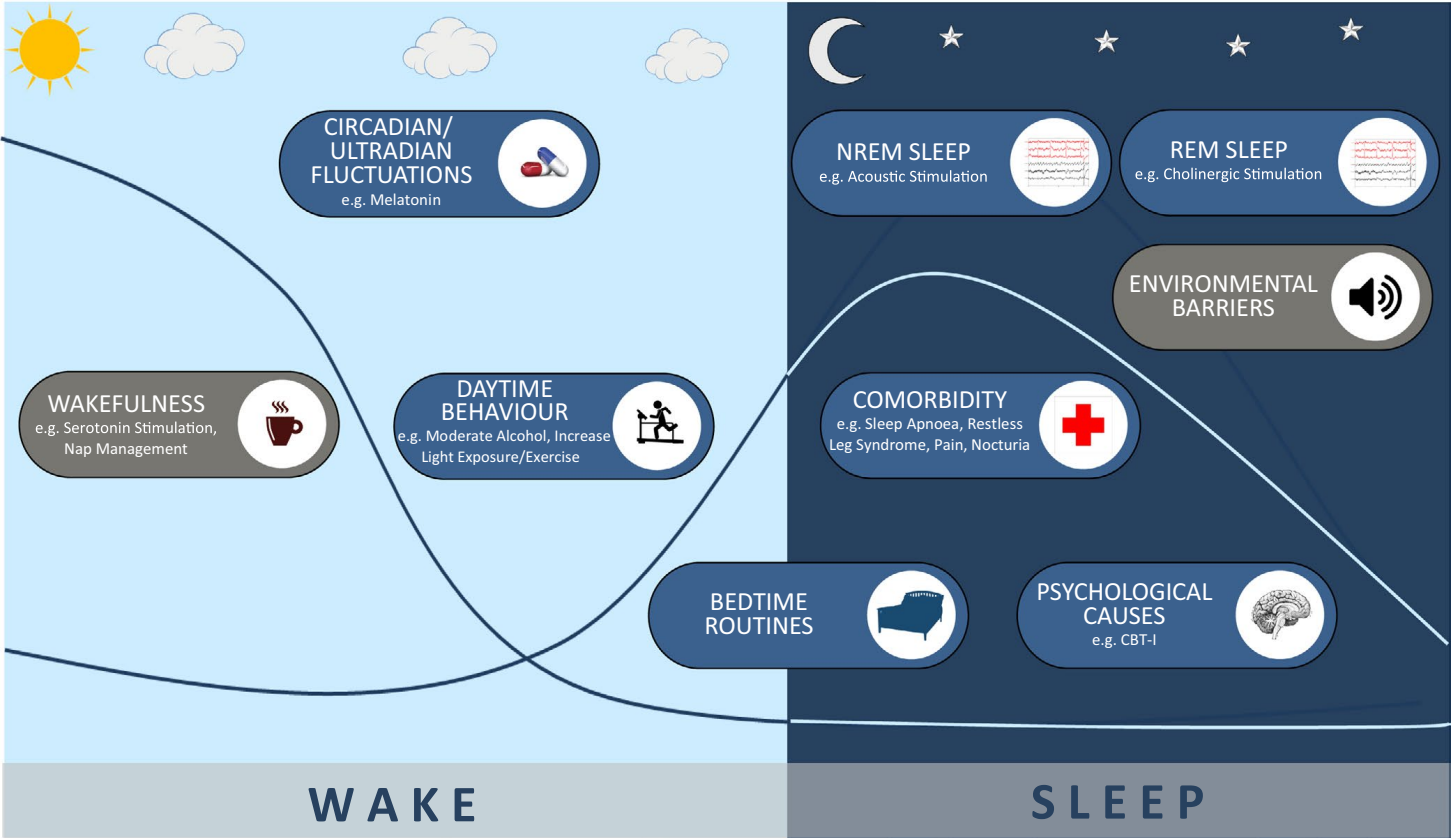
Theoretical interventional strategies to enhance sleep in people developing Alzheimer's disease

There are multiple theoretical targets for sleep enhancement in AD (summarised in Figure 1). Much of the previous literature has focussed on pharmacological interventions (McCleery et al., 2016). Notable disadvantages of this approach, particularly in older adults, include the potential for side effects, interactions and polypharmacy (Maher et al., 2014). Hence, interest in non‐pharmacological sleep‐modifiers is intensifying with recent technological advances permitting exploration of novel approaches such as closed loop stimulation of slow wave sleep through sound or electrical brain stimulation (Ngo et al., 2013) and glasses to deliver Bright Light Therapy (Sekiguchi et al., 2017). Lifestyle interventions are often cost effective and non‐toxic and have the potential to enhance sleep in early stages of AD, but have not previously been the focus of reviews in mild AD and MCI. We include all potential types of intervention, including lifestyle interventions, in the searches for this review.

Here we systematically review the literature on sleep interventions for people meeting established criteria for either Mild Alzheimer's Disease or Mild Cognitive Impairment. We focus specifically on AD rather than other subtypes of dementia as there are specific mechanisms proposed to link sleep disturbance to AD. In the absence of trials of sufficient length or size to establish the efficacy of sleep disturbance treatment on AD disease progression, we first review studies that measure the effect of intervention on sleep as an initial step in the translational pathway to slowing AD progression. This review does not explore the effect of interventions on specific sleep phenotypes due to the paucity of data. Improving sleep regardless of presence or absence of sleep disorder could plausibly impact on AD. Therefore inclusion and exclusion of any underlying sleep disorders in study design was noted, permitted and discussed.

In order to assess the degree to which different avenues for treating sleep in early AD have been explored, we, a priori, created a logic model to incorporate different possible intervention targets (Figure 1). We then compare the scope of existing studies with the model to guide where gaps might lie. The 9 targets included; promotion of wakefulness (to build sleep pressure), optimising circadian fluctuations, improving bed time routines (to remove barriers to sleep), removing overnight environmental barriers to sleep, enhancing Non‐Randomised Eye Movement (NREM) sleep, improving REM sleep, treating psychological causes of insomnia, optimising day time behaviour to promote sleep at night and treating comorbidities that interrupt sleep.

## 2 METHODS

The review followed recommendations by the Preferred Reporting Items for Systematic Review and Meta‐Analyses Statement (PRISMA) (Moher et al., 2009). The protocol was registered in the Prospero Database (registration number CRD42019126320).

### 2.1 Eligibility criteria

All peer reviewed articles written in English were eligible for inclusion. The following criteria were used:

#### 2.1.1 Participants

Inclusion criteria:

1. Adults aged greater than 18 (limit set to avoid excluding studies in genetic dementias); and
2. Male or Female; and
3. At least 75% of the studied group meet the following criteria:

Exclusion criteria:

- Satisfies established diagnostic criteria for MCI e.g. Albert Criteria (Albert et al., 2011), Peterson Criteria (Petersen et al., 1999) or would be expected to meet these criteria if the study was conducted before 1999.

Or:

b Satisfies established diagnostic criteria for AD dementia e.g. DSM‐IV (American Psychiatric Association, 2000), DSM‐V (American Psychiatric Association, 2013), ICD‐10 (World Health Organization, 2004), ICD‐11 (World Health Organization, 2020), NINCDS‐ARDA (McKhann et al., 1984), or would be expected to meet these criteria if the study was conducted before 1984.

1. In order to focus on mild AD and MCI we excluded studies where a majority of the studied group is likely to have Moderate to Severe AD dementia as evidenced by:

- Mini Mental State Examination (MMSE) less than 20;

Or:

b Clinical Dementia Rating (CDR) greater than or equal to 2;

Or:

c Equivalent measure.

#### 2.1.2 Exposure

Interventions of interest include any pharmacological or non‐pharmacological treatment/technique primarily utilised to improve the duration or quality of sleep.

#### 2.1.3 Comparator

The studied intervention must be compared with at least one other intervention, non‐exposure or placebo.

#### 2.1.4 Outcome

Sleep outcomes e.g. total sleep time, sleep efficiency or sleep latency measured by an appropriate validated sleep outcome measure.

#### 2.1.5 Study design

Eligible studies included comparison study designs e.g. randomised controlled trials (RCTs), group trial designs, case‐control, cross‐sectional and prospective cohort studies as well as multiple base studies. Systematic reviews and meta‐analyses were reference tracked. Case reports, abstracts and grey literature were excluded.

### 2.2 Search strategy

Medical Subject Headings (MeSH) index terms were used together with free text to capture relevant results. Preliminary research was undertaken to identify relevant synonymous terminology and refine the search strategy. The full list of search terms is provided in Appendix S1. The following electronic databases were searched: The Cochrane Library, MEDLINE (1946 to present), EMBASE (1974 to present), CINAHL Plus (1937 to present), British Nursing Index (1994 to present) and PsycINFO (1806 to present) and the trial registry WHO ICTRP. Citations of the included papers were hand searched for additional studies. The search was undertaken on 19th August 2019 and repeated on 8th September 2020.

### 2.3 Study selection

Search results were merged using reference management software and duplicate records of the same report removed. A sample of 10% of the total titles were reviewed by each reviewing team member to ensure consensus in decision making. Remaining titles were reviewed individually by a member of the reviewing team in order to exclude studies obviously not meeting criteria.

Abstracts of the retained references were examined against inclusion and exclusion criteria with a further sample of 10% reviewed by each reviewing team member to ensure consensus. The full texts of remaining articles were checked against inclusion/exclusion criteria by two members of the review team. In the process of the search, the reference lists of any identified relevant review articles were hand searched to ensure capture of references not identified on the electronic databases.

Retained studies were recorded in a spreadsheet and included study characteristics, details of the patient/population group, intervention and primary outcomes. Where required, correspondence with investigators was attempted in order to gain further study data or to clarify study eligibility. Discrepancies were resolved through discussion with the first author (JB) in consensus with the reviewing team.

### 2.4 Data extraction

Data extracted from the retained articles (Table 1) included: Participant Characteristics (No. of participants, country, gender, age), Condition/Diagnosis, Sleep Co‐Morbidity, Study Type, Intervention(s) Studied, Intervention Dosage/Duration, Control Group, Validated Outcome Measure Used, Sleep Parameters Measured, Data on Efficacy, Data on Complications.

**Table 1:** Individual Study Characteristics

Author/Year	Condition/ Diagnosis	Sleep Co‐Morbidity Status	Country	No. Total (Female)	Mean Age (*SD*)	Study Type	Intervention	Treatment Dosage/ Duration	Control	Validated Outcome Measure	Sleep Parameters Evaluated
Cassidy‐Eagle et al. (2018a) and Cassidy‐Eagle et al. (2018b)^a^	MCI	Insomnia (DSM IV Criteria)	United States	27 (‐)	89.4	Randomised Controlled Trial	Cognitive Behavioural Therapy ‐ Insomnia	Six 1‐hr sessions	Active control class	Wrist Actigraphy ISI	TST, SE, WASO, SL ISI Total
Cooke et al. (2006)	Mild to Moderate AD	Sleep/ OSA Co‐Morbidities Excluded	United States	76 (25)	IG1 77.8 (7.4) IG2 77.6 (11.5) IG3 79.8 (8.3) CG 78.3 (6.2)	Prospective Study	Donepezil Galantamine Rivastigmine	On stable dose of AChE Inhibitor for 2 months	Non Medication Arm	Polysomnography	TST, SE, WASO, SP, %REM, %N1, %N2, %N3/N4, TIB, PLMI, AI, AH
Cooke, et al. (2009))	Mild to Moderate AD with OSA	OSA only	United States	52 (13)	77.8 (7.3)	Randomised Controlled Trial	CPAP	Therapeutic CPAP for 3 weeks in intervention arm Therapeutic CPAP for 3 weeks in both arms	Placebo CPAP	Polysomnography	TST, SE, WASO, SL, SP, %REM, %N1, %N2, %N3/N4, TIB, AI
Cooke, et al. (2009))	Mild AD with OSA	OSA only	United States	10 (3)	75.7 (5.9)	Prospective Study	CPAP	Continued use’ over approximately 3 years	Non Intervention Arm	PSQI ESS FOSQ	PSQI Total ESS Total FOSQ Total
Cruz‐Aguilar et al. (2018) and Cruz‐Aguilar et al. (2020)^b^	Mild to Moderate AD	‘Alteration to Sleep Pattern’ determined clinically	Mexico	8 (‐)	65.6 (2.9)	Single Blind Placebo Controlled Crossover Study	Melatonin	5 mg nocte for 1 day	Placebo crossover for 1 day	Polysomnography	SL, NREM SL, NREM Relative Power, NREM EEG Coherence
Fultz et al. (2019)	Mild to Moderate AD	Insomnia (DSM V Criteria)	United States	285 (186)	IG 69.6 (8.7) CG 69.1 (8.5)	Randomised Controlled Trial	Suvorexant	28 day total period. 10 mg nocte for 14 days escalated to 20 mg nocte if insufficient response	Placebo Arm	Polysomnography	TST, SE, WASO, SL, %REM, %N1, %N2, %N3/N4, AI, REM SL
Jean‐Louis et al., 1998	MCI and Mild AD	Self reported sleep‐wake disturbance	United States	10 (6)	68.8	Double‐Blind Crossover Trial	Melatonin	6 mg nocte for 10 days	Placebo Arm	Wrist Actigraphy	TST, SE, WASO, SL, Transition, TWT
Kouzuki et al. (2020)	MCI and Mild AD	Mixed Population ± Sleep Disorder	Japan	35 (18)	IG1 76 IG2 78 IG3 82	Randomised Controlled Trial	Aromatherapy (Aroma Oil as Bath Salt)	3 armed trial with 0.1%, 0.5% and 1% strengths	Multiple Treatment Arms	PSQI ‐J	PSQI TST, SE, SL, Total, Daytime Dysfunction, Sleep Disturbances, Subjective Sleep Quality
Ladenbauer et al. (2017)	MCI	Participants with significantly reduced SWS excluded	Germany	16 (7)	71 (9)	Randomised Crossover Trial	Transcranial Stimulation	Anodal current applied via electrodes at frontal locations F3 and F4, oscillated sinusoidally at 0.75 Hz [0–265 µA)	Sham Stimulation	Polysomnography	TST, WASO, %REM, %N1, %N2, %N3/N4, EEG Slow Oscillation and Spindle Characteristics, Spectral Power
Markowitz et al. (2003)	Mild to Moderate AD	Mixed Population ± Sleep Disorder	Canada New Zealand South Africa UK USA Belgium	261 (157)	IG 75.3 (7.5) CG 74.6 (7.6)	Randomised Controlled Trial	Galantamine	12 mg bd	Placebo Arm	PSQI	PSQI TST, SE, SL, Total, Daytime Dysfunction, Sleep Disturbances, Subjective Sleep Quality
Moraes et al. (2006)	Mild to Moderate AD	Moderate/ Severe Sleep Disorders excluded	Brazil	35 (24)	IG 77.4 (6.6) CG 74.5 (9.8)	Randomised Controlled Trial	Donepezil	5 mg od for 1 month increasing to 10 mg od	Placebo Arm	Polysomnography EEG Spectral Analysis	TST, SE, SL, %REM, %N1, %N2, %N3/N4, PLMI, AH, REM Characteristics
Naharci et al., (2015)	AD and Mixed Dementia	Mixed Population ± Sleep Disorder	Turkey	78 (47)	IG1 81.5 (6.9) IG2 80.9 (7.4) IG3 79.1 (7.7) CG 77.4 (66.3)	Prospective Study	Donepezil Galantamine Rivastigmine	5−10 cm^2^ od 5−10 mg od 8, 16, 24 mg od	Healthy Population	PSQI	PSQI Total
Naismith et al. (2018)	MCI	Primary Sleep Disorder excluded	Australia	35 (19)	IG1 69.4 (9.5) CG 70.0 (8.8)	Randomised Controlled Trial	“Sleep Well, Think Well” Group Program	8 week group program (Four 1 hr face to face sessions and four telephone session)	Non‐directive Control Group	PSQI ESS Wrist Actigraphy	TST, SE, WASO PSQI Total ESS Total
Papalambros et al. (2019)	MCI	Circadian Rhythm Disorders/ OSA excluded	United States	9 (5)	72	Randomised Crossover Trial	Phase Locked Loop Acoustic Stimulation	Parameters for stimulation as per previous established algorithm (Santostasi et al., 2016)	Sham Stimulation	Polysomnography	TST, SE, WASO, SL, %REM, %N1, %N2, %N3/N4, AI, Spindle Characteristics
Petit et al. (1993)	Mild to Moderate AD	Mixed Population ± Sleep Disorder	Canada	8 (3)	61.9	Double‐Blind Crossover Trial	Tetrahydroaminoacridine (THA)	50 mg od for 1 week 75−100 mg od for subsequent week	Placebo Period	Polysomnography	SE, SL, %REM, %N1, %N2, %N3/N4, REM Characteristics
Wang et al, (2020)	MCI	Mixed Population ± Sleep Disorder	China	111 (68)	IG 68.4 (5.3) CG 68.2 (5.1)	Randomised Controlled Trial	Structured Limbs Exercise Program	Three 60 min exercise sessions per week for 12 weeks. Two Health Promotion Classes over 12 weeks	Health Promotion Classes only	PSQI	PSQI Total

Abbreviations: AH, Apnoea Hypopnoea Index; AI, Arousal Index; CG, Control Group; DSM, Diagnostic and Statistical Manual; ESS, Epworth Sleepiness Scale; FOSQ, Functional Outcomes of Sleep Questionnaire; IG, Intervention Group; ISI, Insomnia Severity Index; NINCDS‐ADRDA ‐ National Institute of Neurological and Communicative Disorders and Stroke and the Alzheimer's Disease and Related Disorders Association; OSA, Obstructive Sleep Apnoea; PLMI, Periodic Limb Movement Index; PSQI, Pittsburgh Sleep Quality Index; REM, Rapid Eye Movement; SE, Sleep Efficiency; SL, Sleep Latency; TIB, Time in Bed; TST, Total Sleep Time; TWT, Total Wake Time; WASO, Wake After Sleep Onset.

^a^ 2 separate papers written by Cassidy‐Eagle reporting data from the same study. Analysis was focussed on paper reporting sleep outcome measures in more detail to avoid over‐representation of data. Quality appraisal and risk of bias assessment identical due to same study design.

^b^ 2 separate papers written by Cruz‐Aguilar reporting data from the same study. Analysis focussed on combined findings. Quality appraisal and risk of bias assessment identical due to same study design.

### 2.5 Quality assessment

Methodological quality of eligible studies was evaluated using the Joanna Briggs Institute Checklist for Quasi‐Experimental and Randomized Controlled Trials (Tufanaru et al., 2017). Independent evaluation of quality was undertaken by two reviewers (SH and JB) and consensus reached jointly.

Risk of bias for all included studies was also assessed using the Cochrane Collaboration's Tool for Assessing Risk of Bias (Higgins et al., 2019). Two reviewers (SH and JB) undertook this analysis independently before reaching joint consensus.

### 2.6 Data synthesis and analysis

Due to substantial heterogeneity in outcome measures no meta‐analysis of quantitative data was appropriate. Therefore a narrative synthesis of included studies was performed focusing on population characteristics, interventions utilised and outcomes. The main focus of analysis was on the performance of the intervention assessed against a comparison intervention or control.

## 3 RESULTS

### 3.1 Included studies

**Figure 2:**
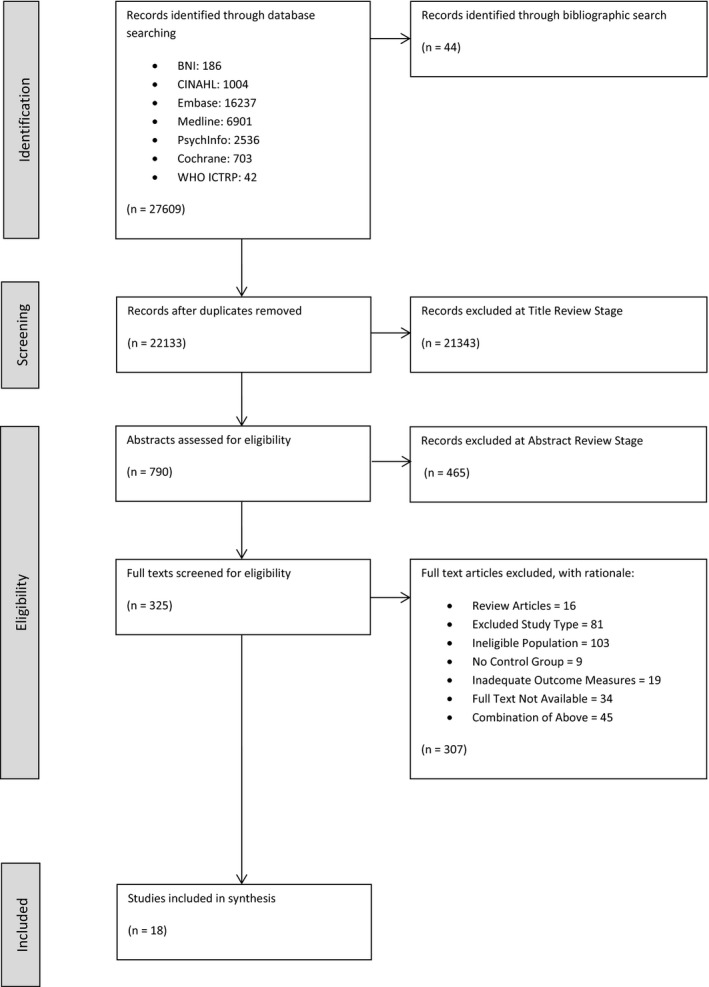
PRISMA flowchart

The search strategy identified 27,609 unique records alongside an additional 44 identified through bibliographic search. 790 abstracts and subsequently 325 full text articles were screened for eligibility with a total of 18 articles selected for inclusion which were published between 1998 and 2020 (see PRISMA flowchart – Figure 2). There were two predominant reasons for exclusion, (a) ineligible study type e.g. intervention not analysed against an alternative or control, (b) ineligible population e.g. due to severity of dementia. The 18 articles identified reported on data from 16 separate studies. Attributes of individual included studies are listed in Table 1.

### 3.2 Participants and comparison measures

A summary of combined included study characteristics is shown in Table 2.

**Table 2:** Combined included study characteristics

Demographics	
Total Participants (% Female)	1,056 (56.9)
Mean Age	73.5
Diagnosis and Severity	
Cognitive Diagnostic Criteria Utilised	NINCDS‐ADRDA (*n* = 8) Peterson MCI Criteria (*n* = 3) Albert Criteria (*n* = 1) DSM‐IV (*n* = 1) DSM‐V (*n* = 1) DSM‐V and Peterson MCI Criteria (*n* = 1) Mayo Clinic Criteria (*n* = 1)
Assessment of Dementia Severity Tool	MMSE (*n* = 10) MoCA (*n* = 2) CDR (*n* = 2) TDAS (*n* = 1) Not reported (*n* = 1)^a^
Baseline Cognitive Scores by Tool	Mean MMSE 22.38/30 (*n* = 9) Median MMSE 28/30 (*n* = 1) Mean MOCA 21.93/30 (*n* = 2) Mean CDR 1.38 (*n* = 2) Mean TDAS 7.57 (*n* = 1)
Sleep Co‐Morbidity Status	Sleep Disorders Included:‐ Mixed Population ± Sleep Disorder (*n* = 5) OSA (*n* = 2) Insomnia by DSM V Criteria (*n* = 2) Clinically determined ‘Alteration to Sleep Pattern’ (*n* = 1) Sleep Disorders Excluded:‐ All (*n* = 2) Self‐Reported Sleep‐Wake Disturbance (*n* = 1) Moderate‐Severe Sleep Disturbance (*n* = 1) Circadian Rhythm Disorders and OSA (*n* = 1) Significantly Reduced SWS (*n* = 1)
Comparison	
Methodology Used	Placebo Groups (*n* = 4) Active Non‐Pharmacological Control Groups (*n* = 3) Non‐Treatment Arms (*n* = 2) Placebo Crossover Designs (*n* = 5) Healthy Population Group (*n* = 1) Multiple Treatment Arms (*n* = 1)
Outcome	
Measurement Tools	Polysomnography (*n* = 8) Wrist Actigraphy (*n* = 4) PSQI (*n* = 6) ESS (*n* = 2) FOSQ (*n* = 1) ISI (*n* = 1)
Outcome Measure (Tool Utilised)	TST (Polysomnography *n* = 6, Actigraphy *n* = 3, PSQI *n* = 2) SE (Polysomnography *n* = 6, Actigraphy *n* = 3, PSQI *n* = 2) WASO (Polysomnography *n* = 5, Actigraphy *n* = 3) SL (Polysomnography *n* = 6, Actigraphy *n* = 2, PSQI *n* = 2) % REM, N1, N2, N3/N4 Sleep Duration (Polysomnography *n* = 7) AI (Polysomnography *n* = 4) PSQI Total (PSQI *n* = 6) Other e.g. PLMI, AH, Transition, TWT, Spindle Characteristics and REM Characteristics (Polysomnography *n* = 4)

^a^ Diagnosis of MCI made utilising peterson criteria.

### 3.3 Outcome measurement and outcomes

As per Table 2, studies utilised a wide range of measurement tools with broad and heterogeneous primary and secondary outcome measures.

Directly comparable group data across key outcome measures was relatively sparse. Only 8 studies reported TST in minutes, one reporting data from afternoon naps and hence not comparable. SE % was similarly provided in only 8 studies comprising 220 participants in intervention groups and 214 participants in control groups across five studies and for 27 patients in crossover designs over three studies. Surrogate measures of TST and SE were provided by two studies utilising PSQI in 162 intervention participants and 121 control participants and 159 intervention participants and 115 control participants respectively. Structural sleep % by stage N1,N2,N3/N4 and REM group means were provided in 250 participants in intervention groups and 198 in control groups across four studies and in 25 participants across 3 crossover studies. Actual Total PSQI Scores were provided in 284 intervention participants and 207 control groups across five studies.

### 3.4 Adverse events

These were described in three studies. Herring et al. (2020) reported 22.5% of participants taking Suvorexant experiencing adverse events compared with 16.1% of participants in the placebo group. One serious adverse event (fall sustaining ankle fracture), judged non‐drug related, was reported in the Suvorexant group resulting in drug discontinuation with somnolence the most commonly reported side effect, 4.2% versus 1.4% (Suvorexant Group vs. Placebo Group). Donepezil was reported by Moraes et al. (2006) to cause mild and transitory side effects including headache and nausea in 3 patients in an intervention of group of 17. Kouzuki et al. (2020) reported minor dermatological side effects following the use of bath salts. No significant adverse events were reported across the remaining included studies.

### 3.5 Quality appraisal and risk of bias

**Figure 3:**
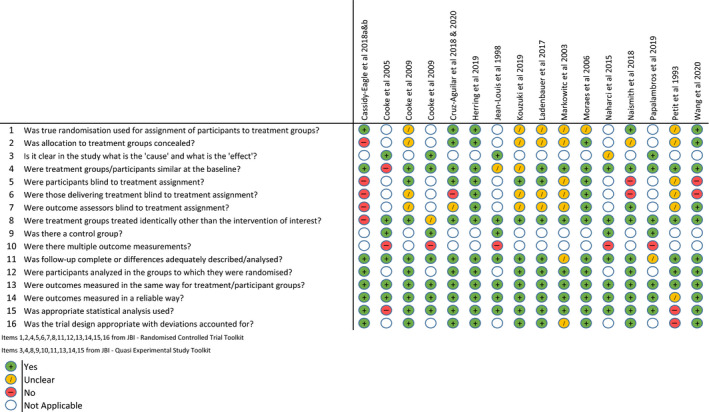
Quality appraisal using Joanna Briggs institute tools by study

**Figure 4:**
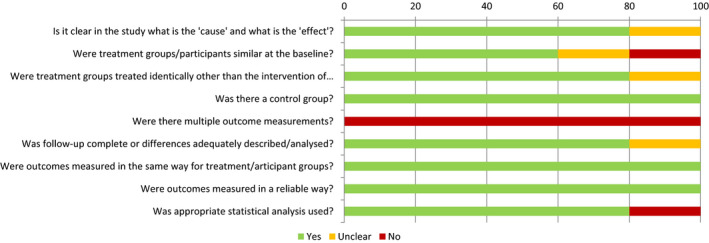
Overall quality appraisal by Joanna Briggs Institute ‐ Quasi experimental tool

**Figure 5:**
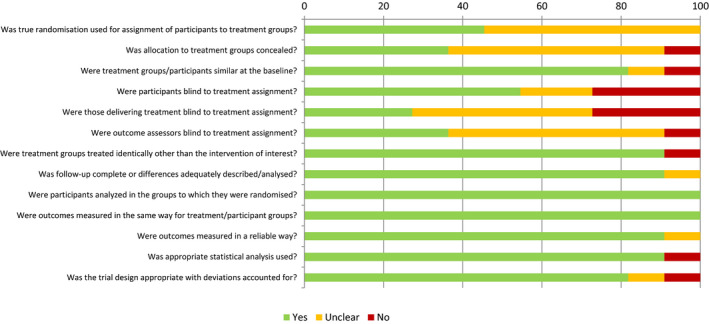
Overall quality appraisal by Joanna Briggs Institute – Randomised controlled trial tool

Quality appraisal was undertaken independently by two reviewers (JB and SH). Inter‐rater reliability calculated using Cohen's Kappa Coefficient was 0.807 [95% CI 0.715–0.900] indicating strength of agreement to be ‘substantial’. For full tabulated results see Figures 3, 4, 5. The overall quality of included studies as measured by the appropriate Joanna Briggs Quality Appraisal Tool was variable. Multiple limitations were identified in one study (Cruz‐Aguilar et al., 2018), however this was partly due to inherent difficulties in concealment and blinding associated with utilising a psychotherapeutic intervention. One further study had 3 limitations identified (Cooke et al., 2006), three studies had two limitations identified (Naismith et al., 2018; Petit et al., 1993; Wang et al., 2020). The remaining eleven studies had 0 or 1 limitation.

**Figure 6:**
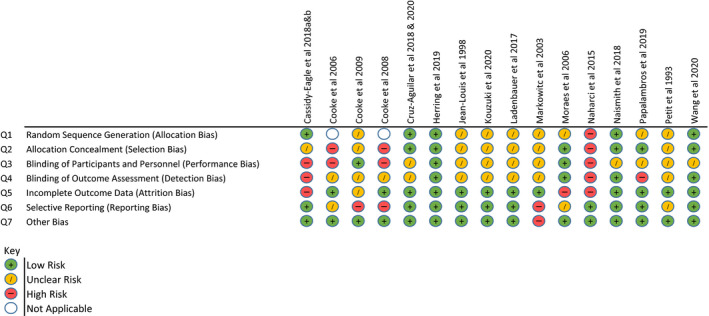
Cochrane collaboration tool for assessing risk of bias

Risk of Bias analysis using the Cochrane Collaboration Tool was also undertaken by two reviewers (JB and SH). Inter‐rater reliability calculated with Cohen's Kappa Coefficient was 0.724 [95% CI 0.613–0.834] also indicating ‘substantial’ strength of agreement. High risk of bias in at least one area was identified in eight included studies with five identified as being at ‘high risk’ in multiple domains. Information regarding group randomisation and blinding was particularly scarce in the majority of studies with only one (Herring et al., 2020) clearly demonstrating low risk in all domains. For full results see Figure 6.

### 3.6 Summary of interventions

Overall we found trials in seven of the nine intervention domains identified in Figure 1. Only one was a large randomised controlled trials powered to find clinical benefit. Most were exploratory, pilot or feasibility trials. There was an over‐representation of trials that used cholinesterase inhibitors to enhance sleep and there were no trials that aimed to promote wakefulness, optimise bedtime routines or remove environmental barriers to sleep. Results of individual trials within each potential intervention domain are as follows:

#### 3.6.1 Enhancing REM

##### 3.6.1.1 Donepezil

Cooke et al. (2006) compared donepezil against galantamine, rivastigmine and a group taking no Acetylcholine Esterase Inhibitor (AChEI). Participants had no prior sleep disorder and analysis was undertaken to test the hypothesis that AchEIs may increase REM sleep and nightmares. Polysomnographic analysis showed a significant decrease in Stage I sleep (*p* = .01) and for those taking donepezil compared to those taking galantamine but no changes compared to other groups. There was also a significant increase in Stage II sleep (*p* = .04) compared to the group taking no AChEI.

However this finding was not replicated when Moraes et al. (2006) tested a similar hypothesis that Donepezil may increase REM sleep percentage and alter the REM sleep EEG power spectrum. Donepezil was directly compared against placebo in a randomized controlled trial of 35 participants with mild to moderate AD and without moderate or severe sleep disorders. No significant differences were found polysomnographically between donepezil and placebo for TST, SE, SL, REM SL or NREM sleep stage percentages. Donepezil was, however, found to significantly increase REM sleep percentage (*p* < .01). Fast Fourier transformation of electroencephalogram (EEG) channel data as part of REM EEG Spectral Analysis also revealed significant decreases in overall theta band power and frontal delta and theta band powers after 6 months of donepezil compared with placebo (0.01 ≤ *p *≤ .04).

Naharci et al. (2015) compared total PSQI prospectively in 78 patients prescribed Donepezil, Galantamine or Rivastigmine and a control group not taking an AChEI with the rationale to evaluate the broad influences of AchEIs on sleep pattern. Study participants included those with and without underlying sleep disturbance. No significant change in total PSQI score was found across groups.

##### 3.6.1.2 Galantamine

Galantamine was found to have little or no impact on sleep architecture by Cooke et al. (2006) in comparison with a group taking donepezil, rivastigmine and a group taking no AChEI. Markowitz et al. (2003) undertook a post‐hoc analysis of a RCT comparing a flexible dose regime of galantamine with placebo. It aimed to explore whether adverse sleep outcomes were associated with galantamine both in participants with and without underlying sleep disorders. Analysis of PSQI and Neuropsychiatry Inventory Sleep Disorder Items revealed no significant difference between groups or from baseline to post intervention. However more recently Naharci et al. (2015) found in a non‐randomised trial that galantamine when compared against donepezil, rivastigmine and a healthy control group significantly decreased total PSQI score (*p* = .028). The highest improvement rate was in those with reported poor sleep quality.

##### 3.6.1.3 Rivastigmine

Rivastigmine was not associated with any change in sleep architecture according to Cooke et al. (2006) although the authors acknowledge that their small sample size (*n* = 8) is likely to have reduced the power to detect a significant effect. Naharci et al. (2015) also found that rivastigmine decreased total PSQI score although this did not reach statistical significance (*p* = .193).

##### 3.6.1.4 Tetrahydroamineacridine – (Tacrine ‐ THA)

The centrally acting anticholinesterase inhibitor THA was compared against placebo by Petit et al. (1993) in a small crossover trial (*n* = 8) involving participants with mild to moderate AD. THA was hypothesised to alleviate sleep disturbance in this group and restore EEG desynchronisation during wakefulness and REM sleep. There were no differences in sleep architecture including SE, SL, SE and percentages of REM and NREM sleep on polysomnographic analysis in the THA versus placebo group. Spectral EEG Analysis by fast Fourier transformations utilising the ratio of power in slow (delta and theta) frequencies to fast (alpha and beta) frequencies revealed REM sleep EEG slowing in the frontal and parietoocciptal regions in the THA group compared to baseline (*p* < .01).

*To summarise,* included studies showed that donepezil may have an impact on sleep architecture through altering the relative proportion of sleep stages, however the precise nature of this effect was not consistently demonstrated. Certainly it has not been shown to improve basic sleep parameters e.g. TST, SE and SL. Galantamine appears to have little effect on sleep architecture (Cooke et al., 2006) but may improve PSQI scores in a small non‐randomised study (Naharci et al., 2015). Again we have found little in the way of replicated findings. Rivastigmine was not demonstrated to have any effect on sleep (Cooke et al., 2006; Naharci et al., 2015). THA may reduce EEG REM sleep cortical slowing as measured by amplitude spectral EEG analysis (Jean‐Louis et al., 1998), although the clinical relevance of this is questionable in a largely withdrawn medication with no demonstrable positive effects on sleep.

#### 3.6.2 Optimising circadian fluctuations

##### 3.6.2.1 Melatonin

A placebo controlled crossover trial of melatonin was conducted by Jean‐Louis et al. (1998) in 10 participants with MCI and Mild AD with self‐reported sleep‐wake disturbance either through difficulty in sleep initiation or frequent nocturnal awakenings. The study aimed to provide objective evidence of its effect on sleep, mood and cognition in the context of previously conflicting evidence.

Actigraphy revealed melatonin to have a statistically significant effect on sleep latency (melatonin group = 14.7 min [*SD* 7.21], placebo group = 26.08 min [*SD* 10.97], *p* = .018). There was also a significant reduction in number of transitions from sleep to wakefulness (melatonin group = 17.75 [*SD* 7.79], placebo group = 31.20 [*SD* 11.37], *p* = .011). There was no significant effect on SE, TST, WASO or phase shift in rest‐activity pattern.

A reduction in sleep latency was also demonstrated in a small pilot study of 8 participants by Cruz‐Aguilar et al. (2018) utilising polysomnography. This single‐blind placebo‐controlled crossover study was conducted over three nights with a habituation night followed by placebo or melatonin, subsequently reversed for the final night. All participants were determined to have had an ‘alteration to sleep pattern’ as determined by a clinical interview with a caregiver. Mean sleep latencies reduced from 34.75 min [SE 8.54] in the placebo group to 15.25 min [SE 2.10] in the melatonin group (*p* = .04).

##### 3.6.2.2 Suvorexant

Suvorexant is a potent dual orexin receptor (OX1R and OX2R) antagonist promoting sleep through inhibition of Orexin A and B, neuropeptides associated with maintenance of wakefulness. Previous studies including Herring et al. (2016) had demonstrated its efficacy in treating insomnia in healthy adults and the elderly population. Herring et al. (2020) assessed its performance in individuals with probable AD and insomnia in a high‐quality, large randomised controlled trial of 285 participants. Screening, baseline and post‐treatment polysomnography was utilised for collection of the primary outcome data (TST) and secondary outcome measures (including WASO, SE, NA, AI and PSG sleep architectural measures). Subjective measures included partner‐rated assessments, Sleep Disorders Inventory Total Score (Tractenberg et al., 2003), the Neuropsychiatric Inventory (NPI) (Cummings et al., 1994), the Clinician's Global Impression of Severity of Patient's Insomnia Score (CGI‐S) (Busner & Targum, 2007).

TST increased in the Suvorexant group by an additional mean of 28.2 min over placebo [95% CI 11.1–45.2], *p* = .001. The number of participants with a greater than 50 min improvement in TST was also significantly greater in the Suvorexant group versus placebo (62% versus 45%, OR 2.2 [95% CI 1.3–3.6], *p* = .002. WASO decreased by 45 min in the Suvorexant group, an additional 16 min when compared to placebo [95% CI −28 to −3], *p* < .05. This effect appeared particularly marked in the final third of the night with an additional decrease of 11 min in the Suvorexant group [95% CI −19 to −3], *p* = .009. SE also increased significantly in the Suvorexant group by an additional 5.7% over placebo [95% CI 2.2–9.3], *p* = .002. There were no significant differences in other polysomnographic secondary endpoints including SL, SL REM, NAW, AI, REM and NREM %. Subjectively, CGI‐S score (*p* = .010) and partner rating of patient's sleep quality (*p* = .021) improved nominally with no significant corresponding change in NPI or SDI.

Subgroup analysis revealed that the effect of Suvorexant on TST was significant only in the mild (21–26) MMSE category of participants (79.3% overall) as opposed to the moderate (12–20) MMSE category (20.7% overall).

#### 3.6.3 Enhancing NREM

##### 3.6.3.1 Phase locked loop acoustic stimulation

Nine participants with Amnestic MCI were recruited for a randomised crossover sham‐controlled study conducted by Papalambros et al. (2019) in order to examine the feasibility of acoustic stimulation during overnight sleep in enhancing Slow Wave Activity (SWA). Participants had no evidence of a circadian rhythm disorder or moderate‐severe sleep apnoea. Acoustic Stimulation was delivered as per a previously established protocol^40^ for one night on two separate occasions seven days apart.

There were no statistically significant effects on basic sleep parameters e.g. TST, SE, SL, WASO or in sleep staging characteristics. Spectral analysis revealed that Slow Oscillation (SO) Activity and Slow Wave Activity (SWA) both increased during Stim ON intervals compared to Sham ON intervals by 15.1% (2.8) and 11.4% (2.9) respectively *p* < .01. A similar decrease in SO and SWA was observed comparing Stim OFF with Sham OFF of 8.1% (3.3), *p* = .040 and 6.6% (2.9), *p* = .046 respectively. Within the same night, Stim ON appeared to increase SO by 22.2% (5.1), *p* = .003 and SWA by 17.9% (4.12), *p* = .004 when compared with Stim OFF. However overall, there was no difference in mean NREM SWA between stim and sham conditions. There were no statistically significant effects on spindle characteristics or on N2, N3 duration.

##### 3.6.3.2 Transcranial stimulation

Ladenbauer et al. (2017) recruited 22 patients with MCI to undergo slow‐oscillatory weak transcranial direct current stimulation (so‐tDCS) during an afternoon nap in a balanced crossover design with stimulation and sham condition. The study was designed to test the hypothesis that this may modulate cortical slow oscillations and thalamocortical spindle activity implicated with memory formation and that this may accordingly improve declarative and procedural memory. Stimulation electrodes were placed bilaterally at frontal locations F3 and F4 with anodal current applied by a battery stimulator. Sleep outcome measures were obtained through PSG/ EEG analysis.

16 participants completed the full experiment. SO power was increased in the so‐tDCS group in frontal (*p* < .001) and centroparietal (*p* = .006) regions. Fast spindle power (12‐15Hz) was significantly enhanced by so‐tDCS in frontal (*p* < .001) and centroparietal (*p* = .041) regions. Additionally slow spindle power (8–12 Hz) was significantly increased after so‐tDCS (*p* = .001). Analysis of phase amplitude coupling concluded that spindle power amplified during SO up‐phases during so‐tDCS leading to stronger synchronisation between SO and spindle power, and marginal improvement in visual memory. So‐tDCS was associated with a significantly greater proportion of time was spent in NREM‐2 (mean difference 18.9%, [95% CI 3.2–34.5], *p* = .021), however no significant differences in sleep time or percentage of remaining sleep stages was found.

##### 3.6.3.3 Melatonin

As well as begin studied in the context of optimising circadian rhythm, the effect of melatonin on NREM sleep was explored by Cruz‐Aguilar et al. (2020) in a further analysis of data from the same study described in Cruz‐Aguilar et al. (2018). Following melatonin administration, mean sleep latencies to all stages of NREM decreased significantly, NREMS‐1: Placebo = 36.63 min SE [7.37], Melatonin = 12.18 min [SE 1.07], *t* = 3.66, *p* = .008), NREMS‐2: Placebo = 44.62 min [SE 8.03], Melatonin = 21.06 [SE 1.82], *t* = 2.86, *p* = .02, NREMS‐3: Placebo = 176.12 min [SE 6.28], Melatonin = 84.31 [SE 4.54], *t* = 13.40, *p *= <0.001. The study provided some support for the hypothesis that melatonin would be associated with increased generation and coherence of slow EEG bands and a decrease in fast EEG band relative power (RP) and coherence. Following adjustment for multiple comparison, during NREMS‐2, a significant increase in theta band (4–7 Hz) RP at C3‐A1 was observed in the melatonin group (*t* = −5.49, *p* = .0009) along with a decrease in gamma band (31–50 Hz) F7‐T3 and F8‐T4 interhemispheric coherence (*t* = 4.18, *p* = .0004). During NREMS‐3, a significant decrease in beta 1 (14–19 Hz) and beta 2 (20–30 Hz) RP was observed at C4‐A2 (*t* = 4.70, *p* = .002) and (*t* = 3.62, *p* = .008) respectively. There was a significant increase in C3‐A1 and C4‐A2 interhemispheric coherence of slow bands; delta (*t* = −3.64, *p* = .008) and theta (*t* = −3.93, *p* = .005) but also of gamma (*t* = −5.13, *p* = .001) and a decrease in beta 1 coherence (*t* = 3.91, *p* = .005). There was also a decrease in all EEG band F7‐T3 and F8‐T4 interhemispheric coherence following melatonin administration. The authors conclude that shorter NREMS onset following melatonin administration in participants with mild‐moderate AD is associated with the RP and EEG coherence differences described above and that these are likely to have been achieved through GABA‐A modulation suggesting that these pathways may be well‐preserved in this patient group.

#### 3.6.4 Treating co‐morbidities

##### 3.6.4.1 Continuous Positive Airway Pressure (CPAP)

Patients with Obstructive Sleep Apnoea (OSA) but no prior diagnosis of a sleeping disorder and AD were recruited to a RCT by Cooke et al. (2009a). The effect of CPAP on sleep was compared with placebo CPAP using polysomnography. Those receiving therapeutic CPAP after one single night had significantly decreased Stage I sleep (*p* = .04) and significantly increased Stage 2 Sleep (*p* = .02). There were no changes in % REM, Stage III, IV Sleep, WASO, SP, TIB, SE or SO in either group. Paired analysis after 3 weeks showed that there were decreases in mean WASO (*p* = .005), TIB (*p* = .002), SP (*p* < .001), Arousals per Hour (*p* = .005) and % Stage I Sleep (*p* = .001) and a significant increase in Stage III Sleep (*p* = .006). In summary CPAP was reported to deepen the level of sleep immediately and later lead to more consolidated sleep with fewer night‐time awakenings and improved SE.

Cooke et al. (2009b) also undertook analysis of the potential longer term effects of CPAP on sleep comparing 5 patients who had continued CPAP (CPAP+) following a previous randomized trial against 5 patients who had discontinued CPAP (CPAP−) approximately 3 years after the initial study. The CPAP+ group were found to have a significant statistical improvement in PSQI while the CPAP‐ group showed a statistical deterioration (effect size = 1.8, *p* < .05). At baseline neither CPAP + nor CPAP− groups had evidence of daytime somnolence as measured by the Epworth Sleepiness Scale (ESS). However at follow‐up, ESS in the CPAP− group had deteriorated in keeping with significant daytime somnolence (effect size = 0.8) whilst ESS in the CPAP+ group remained stable compared to baseline.

#### 3.6.5 Bedtime routines

##### 3.6.5.1 Aromatherapy (Aroma Oil as Bath Salt)

Kouzuki et al. (2020) conducted a randomised controlled trial of aroma oil as a bath salt, to determine its effect on cognition, olfactory function and subjective measures of sleep as determined by PSQI‐J (Japanese version of PSQI). 35 participants with MCI or AD were randomised to three strengths of aroma oils (0.1%, 0.5% and 1%) with bathing performed nightly after 18:00 hr for 24 weeks. No significant changes in PSQI‐J were observed during the study period in all groups.

#### 3.6.6 Daytime behaviour

##### 3.6.6.1 A structured limbs‐exercise program

Wang et al. (2020) conducted a randomised controlled trial of a structured limbs‐exercise program in participants with MCI. The aim was to test for a predicted beneficial effect on cognitive function and to determine the presence and strength of mediating pathways including sleep. 116 participants were randomly assigned to an intervention group consisting of three 60 min structured limbs exercise sessions for 24 weeks (supervised for 12 weeks and then unsupervised for the remaining 12 weeks) or health promotion classes only. PSQI was used as a subjective measure of sleep quality. 111 participants completed the study following 5 exclusions. Total PSQI scores were non‐significantly lower in the intervention group 12 weeks post baseline and subsequently significantly lower 24 weeks post baseline with mean difference −2.45 [95% CI −3.509, −1.391], *p* = .001. A corresponding decrease in depressive symptoms as measured by the Geriatric Depression Scale (GDS) was also reported in the intervention group with a mean difference of −1.64 [95% CI −2.658, −0.613], *p* = .002. In further analysis of mediating effects, sleep quality appeared more strongly related to general cognitive function and had the greater mediating effect when compared to depressive symptoms.

#### 3.6.7 Treating psychological factors causing insomnia

##### 3.6.7.1 Cognitive Behavioural Therapy – Insomnia (CBT‐I)

Cassidy‐Eagle et al. (2018a, 2018b) employed a two‐arm RCT to explore effects of CBT‐I on a sample of 28 participants with MCI and a DSM‐IV diagnosis of insomnia. A standard CBT‐I protocol over 6 weeks was adapted for participants with MCI through a reduction in content covered, inclusion of brief, focused rationales for treatment, time allowed for review and repetition of content as well as reminder/ troubleshooting calls between intervention sessions. It was compared against an ‘Active Control Nutrition Class’ with identical in‐person and phone contact hours which provided learning modules delivered by a dietician covering the impact of diet on aging. These adaptations to standard CBT were hypothesised to improve both objective actigraphy variables and self‐reported insomnia symptoms through the ISI.

CBT‐I was shown to have a highly significant desirable effect on 4 out of 5 outcome variables. ISI decreased from 15.29 (*SD* 2.33) to 3.25 (*SD* 2.05) in the intervention group, SL from 11.03 (*SD* 11.69) to 1.93 (*SD* 3.57), WASO from 104.24 (*SD* 38.21) to 46.95 (*SD* 25.09) and SE increased from 0.792 (*SD* 0.0744) to 0.8804 (*SD* 0.0719). Multilevel regression analysis revealed these outcomes as statistically significant with large effect sizes ISI (*p* < .001 Cohen's *d* ^2^ −4.22), SL (*p* < .001 Cohen's *d* ^2^ −1.73), WASO (*p* < .001 Cohen's *d* ^2^ −2.32), SE (*p* < .001 Cohen's *d* ^2^ 1.89). TST, however, appeared to decrease from 436.61 (*SD* 51.53) to 379.50 (*SD* 75.13) in the intervention group, also finding statistical significance with a large effect size (*p* = .02 Cohen's *d* ^2^ −2.32).

##### 3.6.7.2 “Sleep Well – Think Well” Group Program

Naismith et al. (2018) evaluated the “Sleep Well – Think Well” Program in a pilot RCT of 35 participants with MCI but without underlying sleep disorders. The program involved provision of five booklets, specifically a relaxation booklet, anxiety and worry booklet, healthy sleep practices and intervention booklet, mindfulness booklet and a booklet detailing helpful/ unhelpful thoughts associated with sleep initiation and maintenance to both active and control groups. The active intervention group received four face‐to‐face sessions consisting of psychoeducation, introduction of a guided individualised treatment plan, monitoring of the treatment plan and a summary and consolidation session. Fortnightly compliance monitoring telephone sessions were also conducted. The control group received fortnightly calls to discuss handbook content but no directive, supportive input as above.

There was evidence of improved subjective sleep through a statistically significant reduction of mean total PSQI score in the intervention group (Mean change −2.4 *p* = .023 Cohen's *d* = 0.83). Analysis of individual PSQI components showed statistically significant improvement in SE (*p* = .049) and daytime dysfunction (*p* = .015). There were no statistically significant changes in objective actigraphy parameters including TST, SE and WASO.

## 4 DISCUSSION

### 4.1 Overall findings

Comprehensive literature search revealed a relative paucity of robust data evaluating sleep interventions and their efficacy in a population with mild AD or MCI. The majority of studies were of small scale and many subject to potential QA limitations and bias. In all of the potential theoretical domains for intervention (Figure 1), there are strategies that have not yet been tried. Additionally with the possible exception of the ‘Sleep Well, Think Well’ trial most studies focussed on one intervention as opposed to a multi‐modal approach.

The largest trial in this review conducted by Herring et al. (2020) showed that the dual orexin receptor antagonist Suvorexant led to significant macro‐architectural changes in sleep including an increase in TST, SE and a reduction in WASO. Of note, improvements in TST significantly favoured Suvorexant only in the subgroup with mild (21–26) MMSE scores and not in the moderate group, highlighting potential differences in treatment efficacy between these groups. However, increased rates of daytime somnolence and adverse events may limit its potential adoption in this population.

The most widely studied intervention were the AChEIs likely due to a combination of theoretical, clinical and pragmatic reasons. Early neurochemical theories of sleep hypothesised Acetylcholine playing a primary role in wakefulness and REM sleep (Jouvet, 1972), further strengthened by later work underlying the muscarinic M2 receptor's role in potentiating REM sleep (Baghdoyan & Lydic, 1999; Watson et al., 2012). Clinically, donepezil has been associated with vivid dreams and insomnia with higher incidences versus placebo reported in multiple studies (Burns et al., 1999; Pratt et al., 2002; Rogers et al., 1998) which alongside their common use in those with early cognitive impairment may have driven further research.

The results of trials included here did not reveal a consistent benefit of AChEIs on sleep and it seems unlikely this class of medications will be pursued further to benefit sleep. The one exception to this could involve new technology for drug delivery to modulate available acetylcholine to better mimic physiological fluctuations in cholinergic activity. Given we know that cholinergic synapses are involved in learning while awake (Huerta & Lisman, 1993) and less so during sleep, perhaps more refined temporal target (e.g. daytime only) could improve nocturnal sleep and memory consolidation. We generally noted that there were no identified studies where a daytime‐only pharmacological intervention, promoting wakefulness or attention, was utilised as a tool to enhance night‐time sleep, and this could be a useful future approach.

In a population with OSA and mild AD/MCI, CPAP does appear to be an effective intervention with significant positive effects demonstrated both in sleep architecture and key sleep parameters. Sleep apnoea affects a large proportion of people aged over 50 (Heinzer et al., 2015), therefore treating sleep apnoea holds great promise to improve quality of life and delay AD progression. Sleep apnoea is sufficiently common that screening should be a routine part of clinical practice in memory clinics ‐ we already know that patients can benefit through treatment of sleep apnoea regardless of whether they have AD. The possibility of treating AD is an added benefit. We also propose that screening and, if required, treatment of sleep apnoea should occur before entry into AD clinical trial of any type. Unless we exclude the impact of sleep apnoea on AD progression, we may be clouding benefit from other therapeutic approaches.

Two studies explored the effect of tailored psychotherapeutic programs designed on principles of Cognitive Behavioural Therapy. Outcomes were mixed, with Naismith et al. (2018) reporting a substantial improvement in subjective sleep quality but observing no significant change in objective actigraphy measures. In contrast, Cassidy‐Eagle et al. (2018a, 2018b) reported significant improvement in 4 out of 5 objective outcome measures including SL, WASO and SE as well as significant improvements in subjective quality (ISI). Of note, however, there were potentially significant differences between the two populations with those recruited to the Cassidy‐Eagle et al. (2018a, 2018b) study requiring a DSM‐IV diagnosis of Insomnia in contrast to the Naismith et al. (2018) study. Critically, both studies highlight the feasibility of this approach in MCI.

Melatonin appeared to significantly reduce sleep latency both when analysed utilising PSG by Cruz‐Aguilar et al. (2018) and utilising actigraphy by Jean‐Louis et al. (1998) who also demonstrated a reduction in overnight transitions from sleep to wakefulness. Both studies were of small numbers however with 8 and 10 participants respectively. Other approaches specific to circadian rhythm modification have not been systematically explored despite evidence that circadian rhythms are disturbed in AD.

Three studies specifically explored interventions attempting to enhance the duration or characteristics of NREM sleep. Phase locked acoustic stimulation appeared to have no effect on basic sleep parameters or sleep‐stage architecture but did appear to increase SWA and SO whilst ‘ON’ without increasing its mean overall when compared to a sham comparison group. Similarly, transcranial stimulation during afternoon naps was shown to increase SO power and fast spindle power whilst also enhancing their synchronisation with no significant effects on sleep‐stage architecture. Melatonin reduced sleep latency to all stages of NREM and was associated with increased generation and coherence of slow EEG bands according to Cruz‐Aguilar et al. (2020).

A structured exercise program in participants with MCI was associated with a statistically significant reduction in PSQI score although there was a corresponding significant decrease in Global Depression Score raising questions as to causality direction. Further evidence is likely to arise from the currently recruiting Dementia‐MOVE randomised controlled trial (Haeger et al., 2020). This intends to explore the effects of a long‐term, 6 month, multicomponent exercise program on brain metabolism together with actigraphic and subjective measures of sleep in participants with early AD.

Overall, aside from two larger trials, the studies were of relatively small scale. The studies included also had differing aims in analysing sleep amongst which symptomatic relief of insomnia, macro and micro‐architectural optimisation of sleep and sleep analysis as an effect mediator were found. The overall lack of available evidence was surprising given the broad range of pharmacological and non‐pharmacological sleep interventions available for use in the general population. Striking omissions include the most common group of pharmacological interventions for insomnia (Z‐hypnotics such as zopiclone) which appear to have no evidence base in this group.

There were multiple interventions such as vitamin B12, risperidone, acupressure, bright light therapy, transcutaneous electric nerve stimulation (TENS) and music interventions that were not included in our review either due to the use of unvalidated sleep outcome measures or because there was no comparison group to allow for meaningful conclusions to be drawn. In four cases the diagnoses of mild AD/MCI could not be confirmed to have been made using recognised criteria.

Lifestyle modification was explored only in the context of psychotherapeutic approaches rather than in isolation. It was with regret that a high quality randomised controlled trial in individuals from a general community setting with reduced MoCA scores, testing the effects of a multimodal tailored lifestyle intervention in improving actigraphic and subjective measures of sleep (Falck et al., 2020) could not be included due to our pre‐determined strict methodology regarding diagnostic criteria. The intervention consisted of sleep hygiene measures followed by the formulation of an individually timed Bright Light Therapy and was shown to have improved measures of subjective but not objective sleep quality. Whilst in established mild‐moderate AD dementia there are concerns about cognitive limitations that might prevent behavioural change, it is our belief that this type of tailored intervention may well be a fruitful route for further exploration, particularly given that cognitive function at this stage often still allows significant adaptation of lifestyle and motivation can be high. Furthermore, pharmacological interventions are inherently associated with potential toxicity and the burden of polypharmacy.

### 4.2 Strengths and limitations

This is the first review we know of to focus on sleep intervention in MCI and Mild AD. A comprehensive electronic database search protocol was performed with a wide range of potential sleep interventions sought. Additionally, we summarized findings on multiple dimensions of sleep outcome data. A process for rigorous justification of all subsequent decisions in terms of inclusion/exclusion was utilised. As with the inclusion criteria for AD dementia/MCI, attempts were made to contact authors in cases of ambiguity.

A significant challenge in this review was in formulating inclusion criteria to ensure that studies involving important, clinically relevant interventions were included but also ensuring that studies with an overly heterogeneous population whose findings may not be applicable to our population of interest were excluded. A threshold of 75% was chosen for participants to have an established diagnosis of AD dementia or MCI. It was further decided to exclude any study where the majority of participants have moderate or severe dementia. Naturally these cut‐offs could be criticised either for diluting the population of interest or for failing to allow for inclusion of relevant studies. We therefore cannot exclude the possibility that there may be important findings left unreported. Included studies reported on a range of diverse outcomes, which whilst individually valid, made comparison across studies challenging. Direct comparison was further hindered by variations in study designs permitting or excluding the presence of underlying recognised sleep disorders amongst participants. Ideally, the effect of interventions on specific sleep phenotypes would have been reviewed, however unfortunately this information was not reported on the scale to allow for meaningful comparison. Nonetheless our decision to include studies regardless of phenotype could be argued to increase real‐world applicability to the clinical population and is not contrary to the central hypothesis that an improvement in sleep regardless of baseline may positively impact on AD progression. Quality appraisal scores across included studies were variable and with one exception, those included were largely subject to at least one domain of significant bias thereby potentially compromising their findings. This though, remains consistent with our assertion that there is a paucity of reliable, large‐scale data for this population. For this present review, the risk of publication and language bias for current literature is also recognised.

### 4.3 Clinical implications and future direction

The lack of rigorous evidence presents a significant future research demand. Those with MCI and mild AD might not respond identically to interventions with more robust evidence in the general population or in those with severe AD dementia. Sleep disturbance may well play a role in triggering or facilitating pathophysiology in AD and is the subject of current, ongoing research. Finding evidence‐based treatments for sleep disturbance in a population before more severe manifestations of AD dementia develop, may therefore even offer the potential to delay the onset of more significant functional impairment. Of note, the included studies had a diversity of aims including symptomatic relief of insomnia, optimisation of macro or micro‐architectural sleep parameters and assessing sleep disturbance as an effect mediator for cognitive impairment. In the future, a more standardised set of outcome criteria may be helpful in allowing efficacy of interventions to be more meaningfully and directly compared. As understanding develops of the precise mechanisms by which sleep contributes to the progression of AD pathology, this may occur naturally.

## 5 CONCLUSION

In the specific population of MCI and mild AD there is a relative paucity of evidence available in supporting efficacious interventions for sleep disturbance, nonetheless positive outcomes have been reported. Psychotherapeutic approaches utilising adapted CBT, melatonin, suvorexant and CPAP for OSA all hold promise. Acoustic stimulation and transcranial stimulation significantly affect specific micro‐architectural characteristics of NREM sleep, the long‐term cognitive and disease modifying effects of which can be theorised but are not known. In our opinion, given the wide health risks of OSA, screening should become routine in memory clinics. Despite behavioural interventions often being low cost and non‐toxic, we found remarkably little work in this domain. This review identifies a significant need to explore multiple, alternative sleep interventions through high quality, comparison experimental studies utilising validated sleep outcome measures.

## CONFLICT OF INTEREST

None.

## Supporting information

Appendix S1

## Data Availability

Data available on request from the authors.
