# Supplementary material for: Pharmacological and non‐pharmacological interventions to enhance sleep in mild cognitive impairment and mild Alzheimer's disease: A systematic review
Source: J Sleep Res. 2020 Dec 2;30(4):e13229. doi: 10.1111/jsr.13229 (PMC8365694; doi:10.1111/jsr.13229)
Supplement: Supplementary file 1 — Appendix S1 [file JSR-30-e13229-s001.docx]

Supplementary Information

Pharmacological and Non-Pharmacological Interventions to Enhance Sleep in Mild Cognitive Impairment and Mild Alzheimer’s Disease: A Systematic Review

Appendix 1 - Search Protocol Terms

Cochrane Library, MEDLINE, EMBASE, CINAHL Plus, British Nursing Index, PsycINFO.

(#1 AND #2 AND (#3 OR #4))

#1 Search ("Sleep" OR "Insomnia")

#2 Search (Alzheim* OR "Mild Cognitive Impairment" OR "MCI" OR "Mild Neurocognitive Disorder" OR "MNCD" OR “Age Associated Memory Impairment” OR “Age-Associated Memory Impairment” OR “AAMI” OR "Age Associated Cognitive Decline" OR “Age-Associated Cognitive Decline” OR  "AACD" OR "Cognitive Impairment No Dementia" OR "CIND" OR "Mild Cognitive Disorder" OR "MCD" OR "Age Related Cognitive Decline" OR "ARCD" OR "NMCI" OR "AMCI" OR "MMCI" OR "SMCI" OR "MCIA" OR "Early AD" OR "Mild AD" OR "Prodromal AD" OR "Prodromal Dementia" OR "Pre-Dementia" OR "Pre Dementia" OR "Early Dementia" OR "Mild Dementia" OR "Minimal Dementia" OR "Mild Cognitive Deficit" OR "Mild Cognitive Dysfunction" OR "Mild Cognitive Decline" OR "Early Neurodegeneration) Sort by: Best Match Filters: Humans

#3 Search ("Pittsburgh Sleep Quality Index" OR "PSQI" OR "Epworth Sleepiness Scale" OR "ESS" OR "Insomnia Severity Index" OR "ISI" OR "Functional Outcomes of Sleep Questionnaire" OR "FOSQ" OR "Athens Insomnia Scale" OR "Medical Outcomes Study Sleep Scale" OR "Sleep Wake Activity Inventory" OR "Jenkins Sleep Questionnaire" OR "Jenkins Sleep Evaluation Questionnaire" OR "Sleep Quality Scale" OR "Time Of Day Sleepiness Scale" OR "Regensburg Insomnia Scale" OR "Bergen Insomnia Scale" OR "Quebec Sleep Questionnaire" OR "Consensus Sleep Diary" OR "Pittsburgh Sleep Diary" OR "Multiple Sleep Latency Test" OR "Maintenance Of Wakefulness Test" OR "Actigrap*" OR "Wrist Activity" OR "Polysomnography" OR "PSG" OR "Accelerometry" OR "Actiwatch" OR "MotionWatch" OR "Fitbit" OR "Sleep EEG" OR "Sleep Electro*") Sort by: Best Match Filters: Humans

#4 Search (((Pharm* OR "Drug Treatment" OR "Circadian Rhythm Modulators" OR "Melatonin" OR "Acetylcholinesterase Inhibitors" OR "AChE Inhibitors" OR "Tacrine" OR "Galantamine" OR "Rivastigmine" OR "Donepezil" OR "Antipsychotic*" OR "Ziprasidone" OR "Olanzapine" OR "Risperidone" OR "Quetiapine" OR "Amisulpride" OR "Sulpride" OR "Z-Drug*" OR "Z-Hypnotic*" OR "Zopiclone" OR "Zolpidem" OR "Zaleplon" OR "Benzodiazepine*" OR "Diazepam" OR "Lorazepam" OR "Nitrazepam" OR "Temazepam" OR "Alprazolam" OR "Anti-Histamine*" OR "Antihistamine*" OR "Promethazine" OR "Chlorphenamine" OR "Hydroxyzine" OR "Trazodone" OR "Mirtazapine" OR "Memantine" OR "Amitriptyline" OR "Dietary Supplements" OR "Diet" OR "Herbal Medicine" OR "Natural Medicine" OR "Complementary Medicine" OR "Alternative Medicine" OR "Traditional Medicine" OR "YI gan san" OR "Gongjin dan" OR "Camomile" OR "Kava" OR "Ashwaganda" OR "Hops" OR "Lemon Balm" OR "Valerian" OR "Phytochemicals*" OR Non-Pharm* OR Nonpharm* OR "Psychologic*" OR "Psychotherapy" OR Behavi* OR "Cognitive" OR "CBT" OR "CBTI" OR "CBT-I" OR "Relax*" OR "Stimulus Control" OR "Hygiene" OR "Light Therapy" OR "BLT" OR "Light Exposure" OR "Phototherapy" OR "Exercise" OR "Acupuncture" OR "Complementary Therapy" OR "Exercise Therapy" OR "Mind-Body Therapies" OR "Massage" OR "Chronotherapy" OR "Continuous Positive Airways Pressure" OR "CPAP")) Sort by: Best Match Filters: Humans

WHO ICTRP

(#1 AND #2)

#1 TITLE : Sleep* or Insom* or Slept

#2 CONDITION : Dement* OR Alzheim* OR MCI OR Cognit* OR Memory OR Neurodeg*
